# A competent bidrug loaded water soluble chitosan derivative for the effective inhibition of breast cancer

**DOI:** 10.1038/s41598-020-60888-5

**Published:** 2020-03-04

**Authors:** Nivethaa E A K, Baskar S, Catherine Ann Martin, Ramana Ramya J, Stephen A, Narayanan V, Lakshmi B S, Olga V. Frank-Kamenetskaya, Subathra Radhakrishnan, Narayana Kalkura S

**Affiliations:** 1 Crystal Growth Centre, Anna University, Chennai, 600025 India; 2 Department of Nuclear Physics, University of Madras, Chennai, 600025 India; 3 Department of Inorganic Chemistry, University of Madras, Chennai, 600025 India; 4 National Centre for Nanoscience and Nanotechnology, University of Madras, Chennai, 600025 India; 5 Department of Crystallography, Institute of Earth Sciences, St. Petersburg State University, St. Petersburg, 199034 Russia; 6 Centre for Biotechnology, Anna University, Chennai, 600025 India; 7 National foundation for liver research, Global hospitals, Perumbakkam, Chennai, 600100 India

**Keywords:** Chemical biology, Materials science

## Abstract

Drug resistance and damage caused to the normal cells are the drawbacks which have limited the use of the existing effective anticancer drugs. Attainment of a steady and extended release by encapsulating dual drugs into biocompatible and biodegradable vehicles is the key to enable the use of these drugs for effective inhibition of cancer. In this study, carboxymethyl chitosan (CMCS), a proficient water-soluble derivative of chitosan has been synthesized using chemical route and used for the delivery of 5-Fluorouracil and doxorubicin individually as well as in combination. Carboxymethylation occuring at –NH_2_ and OH sites of chitosan, has been confirmed using FTIR. EDX and Fluorescence studies elucidate the encapsulation of 5-Fluorouracil and doxorubicin into CMCS. The capability of CMCS to release the drugs in a more sustained and prolonged manner is evident from the obtained release profiles. About 14.9 µg/ml is enough to cause 50% cell death by creating oxidative stress and effectuating DNA fragmentation. Amidst the existing reports, the uniqueness of this work lies in using this rare coalition of drugs for the suppression of breast cancer and in reducing the side effects of drugs by encapsulating them into CMCS, which is evidenced by the high hemocompatibilty of the samples.

## Introduction

Changes in lifestyle like lack of physical activity, increase in the consumption of junk food and increased stress levels due to lack of proper sleep have led to an increase in the number of people being affected by cancer. Among the different types of prevalent cancer, breast cancer is the most often recurring cancer in females, consequently leading to death. The number of women affected worldwide by breast cancer has increased from 1.38 million in the year 2008 to 1.7 million in the year 2012. Similarly, the mortality rate has also increased from 4,58,400 in 2008 to 5,21,900 in 2012. The incidence percentage of breast cancer in south central Asia region, under which India falls has increased by 4.2% and the mortality percentage has increased by 1.5% in the year 2012 when compared to 2008^1,2^. Thus, it is the need of the hour to find effective ways to combat breast cancer. Inspite of the already existent medications and radiation therapy for the treatment of cancer, new approaches are required in order to overcome the side effects of the prevalent ministrations like the death of normal cells^3–5^. So, it is essential to find a method by which the available drugs like 5-Fluorouracil (5-FU) and Doxorubicin hydrochloride (DOX) which have the capability to inhibit the growth of solid tumours^6,7^, can be delivered effectively without causing any damage to the normal cells.

Another hindrance encountered while using 5-FU for the treatment of breast cancer is the development of drug resistance due to the expression of Lipocalin2, thus leading to a decrease in the inhibition of cancer cell growth^8^. This can be subdued by the use of another drug in combination with 5-FU. A combination of drugs (for instance 5-FU with Methotrexate, cisplatin, curcumin) has already been used for the treatment of gastric cancer^9^, salivary gland cancer^10^, pancreatic cancer^11^, ovarian cancer^12^, colon cancer and even breast cancer^13^.

A combination of 5-FU with DOX can be used to effectively curb the problem of drug resistance and to inhibit solid tumors. Doxorubicin hydrochloride has been chosen as it is a fluorescent drug which has been widely used for the treatment of various cancers including breast cancer, as it exhibits different mechanism of action to combat and inhibit several types of cancer. Moreover, the presence of fluorescent DOX can also aid in using the prepared system for *in vivo* imaging.

Similarly, the issue of biocompatibility and the harmful effects of the drugs on normal cells can be overcome by encapsulating the drugs into drug delivery carriers that can lead to the sustained, prolonged and targeted release of the drugs^14^. Biopolymers have been the candidates of choice for this purpose due to the inherent properties they possess^15–19^. Of the biopolymers, the most commonly used polymer is chitosan due to it being biocompatible, biodegradable, non-toxic and the presence of chelating groups for the encapsulation of drugs and other molecules. But one major drawback encountered while using chitosan is its insolubility in water. So, chitosan is functionalized in order to make it water soluble. Among these, CMCS is a water-soluble form of chitosan possessing all the properties of chitosan thus making it useful in a wide range of applications like biosensors, in adsorption, in food industry and in the biomedical field^20^.

In the present study, dual drug (5-FU+DOX) loaded CMCS has been synthesized and characterized. The release of the drugs from the biopolymer has been studied and the release kinetics have also been analyzed. Cytotoxicity of the dual drug loaded system towards MCF-7 has been studied and the IC_50_ value has been obtained.

## Results and Discussion

### FTIR studies

**Figure 1:**
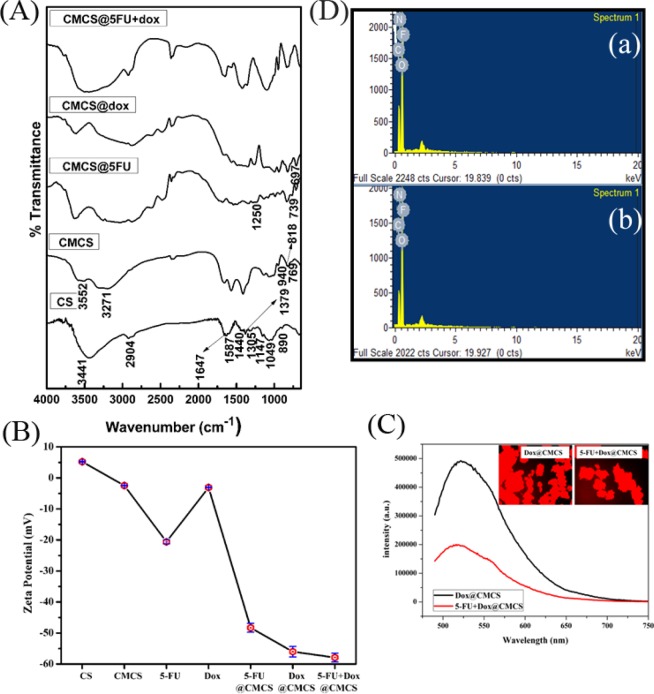
(**A**) FTIR spectrum of chitosan, carboxymethyl chitosan, CMCS@5-FU, CMCS@DOX and CMCS@5-FU+DOX, (**B**) Zeta potential of the drugs and various synthesized compounds, (**C**) Fluorescence spectroscopy of DOX and dual drug encapsulated CMCS with Fluorescence microscopy of DOX incorporated samples as inset and (**D**) EDX spectrum of (a) 5-FU@CMCS and (b) 5-FU + DOX@CMCS.

The FTIR spectrum of CMCS with and without drug encapsulation are shown in Fig. 1(A). As CMCS was prepared taking chitosan (CS) as the precursor, the FTIR spectrum of CS is also presented in Fig. 1(A). All the FTIR spectra show the presence of peaks of NH_2_ twisting at ~890 cm^−1^, C–O stretching at ~1045 cm^−1^, C_3_–O stretching overlapped with C=O stretching at ~1147 cm^−1^, C–O–C stretching at ~1254 cm^−1^, C–N stretching of amide III at ~1305 cm^−1^, C–H bending of –NHCO of amide at ~1360 cm^−1^, CH_2_ bending at ~1440 cm^−1^, peak of NH_3_^+^ at ~1570 cm^−1^ and N–H bending and C=O stretching of amide bonds at 1650 cm^−1^ ^21^. Apart from this the spectrum of CS contains a broad peak at ~3441 cm^−1^ corresponding to N–H and O–H stretching. The FTIR spectrum of CMCS shows additional peaks at C–H oop of aromatics at ~769 cm^−1^, N–H wag at 818 cm^−1^ and peak corresponding to CH_2_COOH at ~1403 cm^−1^ ^22^. Apart from the appearance of these additional peaks a broadening and splitting of the peak of CS present at ~3441 cm^−1^ was also observed. The peak was split into two, one at 3271 cm^−1^ due to the overlapping of asymmetric axial deformation of COO^−^^23,24^ and the other at ~3552 cm^−1^ corresponding to free hydroxyl groups. This splitting may be due to the attachment of few carboxymethyl groups at the OH site. This splitting is also seen in all the drug loaded systems. An evident splitting of NH_3_^+^ and NH_2_ peak is also observed, indicating the attachment of carboxymethyl groups to the amine functionality of CS^25^. The formation of CMCS is also evident from the XRD results (Fig. S1).

In the case of 5-FU encapsulated CMCS, three new peaks were observed at ~727 cm^−1^, 1250 cm^−1^ and 1519 cm^−1^ corresponding to the C–H out of plane deformation of CF= CH, C–N stretching and C–H in plane bending vibration, respectively^26^. Moreover, a slight shift of the peak at ~1660 cm^−1^ and a variation in the intensity of the same peak is observed which is attributed to the interaction of N–H groups of CMCS with the carbonyl group and F moiety of 5-FU^27^. Similarly, deviation in the peak at ~1660 cm^−1^ has been observed on loading doxorubicin into CMCS, apart from the appearance of two new peaks at 1263 cm^−1^ corresponding to C–O–C asymmetric stretch of doxorubicin overlapped with C–O stretch of alcohol and another at 675 cm^−1^ pertaining to out of plane OH bending, showing the encapsulation of doxorubicin on CMCS^28,29^. Likewise, the peaks representing CMCS, 5-FU as well as DOX were present in the case of dual drug loaded CMCS, depicting the encapsulation of both the drugs into CMCS.

### Zeta potential

The zeta potential values obtained for the case of different samples are shown in Fig. 1(B). Pure chitosan exhibits a zeta potential of ~+5 mV whereas, carboxymethyl chitosan shows a zeta potential of ~−2 mV. Apart from this another small peak area is observed pertaining to positive zeta potential. The negative zeta potential is due to carboxymethylation of chitosan and the positive zeta potential peak is due to the presence of a few protonated amine groups like in the case of chitosan. This is indicative of the formation of carboxymethyl chitosan and also of the fact that carboxymethylation does not occur at all -NH_2_ sites. For the case of 5-FU encapsulated CMCS, zeta potential is ~−48 mV, which is high when compared to the value obtained for pure 5-FU. Similarly, for the case of DOX @CMCS the zeta potential is ~−56 mV, which is higher compared to ~−3mV obtained for pure DOX. The zeta potential of dual drug nanocomposite is observed to be ~−57 mV, which is slightly high when compared to the single drug encapsulated systems. An increase in zeta potential is observed for the drug loaded systems when compared to pure CMCS. A small contribution to this increase comes from the crosslinking of the polymer. Another, major reason for this increase is the neutralization of protonated amine groups in the polymer due to the encapsulation of drugs (i.e.) RNH_3_^+^ < RCOO^−^ for drug loaded systems^30,31^. Thus, the drug loaded samples are devoid of the small positive potential observed in the case of CMCS. The zeta potential values of the drug loaded systems show their high colloidal stability which in turn evidences the encapsulation of drugs into CMCS, thus making it a suitable candidate for the delivery of these drug molecules^32^.

### Fluorescence spectroscopy and microscopy

Fluorescence spectroscopy and microscopy were performed to confirm the presence of DOX in the systems as it is the only fluorescent moiety present in the systems (Fig. 1(C)). The samples were excited at 498 nm and the emission peak is obtained at ~530 nm instead of 598 nm which is the emission wavelength of DOX^33^. This shift in the emission wavelength is indicative of the encapsulation of DOX into CMCS. Shifts in the emission wavelength due to the loading of DOX into different systems has been reported before^34^.

Fluorescence microscopy images of DOX loaded CMCS and dual drug loaded CMCS are shown in the inset of Fig. 1(C). The images were taken using red filter. The red color zones in the images evidence the presence of fluorescent DOX in the systems.

### EDX analysis and particle size measurements

The EDX spectrum of 5-FU containing systems are shown in Fig. 1(D). The EDX spectrum obtained in both the cases shows the presence of Carbon, Nitrogen, Oxygen and Fluorine. C, N and O belong to the polymer whereas F comes from 5-FU. The presence of Fluorine confirms the encapsulation of 5-FU into CMCS in both the cases. Fluorine peak was absent for CMCS as well DOX@CMCS elucidating that the presence of F is only due to the inclusion of 5-FU. The surface morphology of the prepared samples was also studied using SEM and the data is presented in Fig. S2.

**Figure 2:**
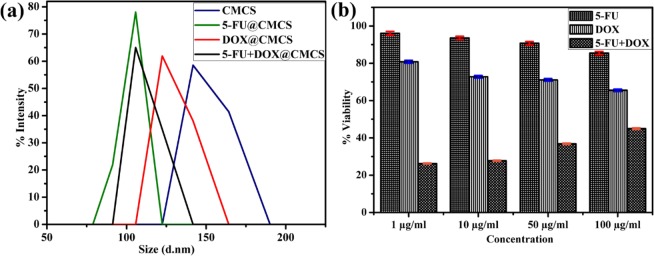
(**a**) Particle size measurents using dynamic light scattering for the various prepared systems. Values are expressed as Mean of 3 independent trials and (**b**) Plot of % viability vs sample concentration for pure drugs (5-FU, DOX and 5-FU + DOX). Values are expressed as Mean ± S.D. of 3 independent experiments.

The size of the particles in all the prepared systems analyzed using dynamic light scattering are shown in Fig. 2(a). The particle size is about 151 + 15 nm, 103 + 8 nm, 129 + 10 nm and 112 + 6 nm for CMCS, 5-FU@CMCS, DOX@CMCS and 5-FU+DOX@CMCS, respectively.

### Evaluation of drug encapsulation and loading

The encapsulation efficiency and loading efficiency were calculated using the standard formula reported previously and has been presented below^35^. The amount of free drug was calculated by measuring the absorbance of the supernatant solution collected, after precipitation of particles while synthesizing the drug encapsulated systems. The encapsulation efficiency of 5-FU for the case of 5-FU@CMCS and DOX for DOX@CMCS are found to be 86% and 81% respectively. The loading efficiency is 27% for 5-FU@CMCS and 13.2% for DOX@CMCS. Similarly, 81% of 5-FU encapsulation and 76% of DOX encapsulation into CMCS has been attained in the case of dual drug loaded CMCS and the loading efficiency of 5-FU and DOX for dual drug loaded CMCS are 23% and 11%, respectively.

$${\rm{Encapsulation}}\,{\rm{efficiency}}=\frac{{\rm{Total}}\,{\rm{drug}}-{\rm{Free}}\,{\rm{drug}}}{{\rm{Total}}\,{\rm{drug}}}\times 100$$

$${\rm{Drug}}\,{\rm{loading}}\,{\rm{efficiency}}=\frac{{\rm{Total}}\,{\rm{Drug}}-{\rm{Free}}\,{\rm{drug}}}{{\rm{Weight}}\,{\rm{of}}\,{\rm{drug}}\,{\rm{loaded}}\,{\rm{nanocomposite}}\,{\rm{taken}}}\times 100$$

### Drug release studies

**Figure 3:**
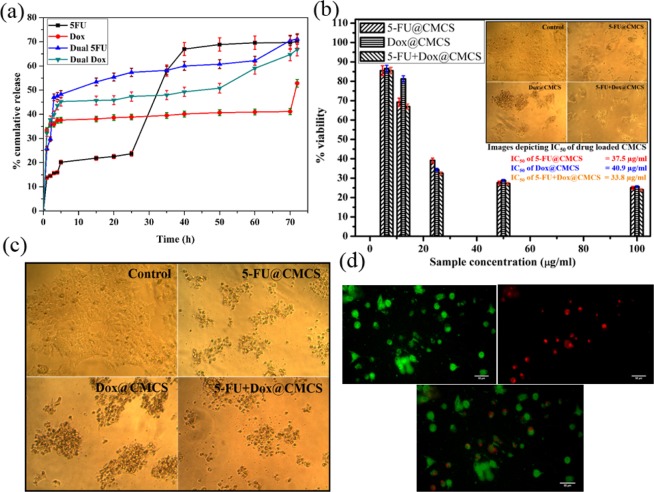
(**a**) *In-vitro* drug release studies showing cumulative percentage drug release from CMCS during single drug encapsulation and dual drug encapsulation in PBS (pH-5) for a time period of 72 h at room temperature, (**b**) Plot of % cell vialbility (*in vitro*) vs sample concentration for single and dual drug encapsulated CMCS with inverted microscope images at IC_50_ concentration as inset, (**c**) Inverted microscope images showing cell viability on addition of 100 µg/ml of drug loaded samples and (**d**) Microscopy images showing AO-PI staining of MCF-7 cells after treatment with 5-FU + DOX@CMCS with a scale bar of 50 µm. Values are expressed as Mean ± S.D. of 3 independent experiments.

The release of the anticancer drugs from CMCS was evaluated at regular time intervals for a period of 72 h and is shown in Fig. 3(a). The phenomenon responsible for the release of the drugs from the polymer matrix has been studied by splitting the release profile into regions and fitting the data using the standard kinetic models. A release of 70% of 5-FU from CMCS, 53% of DOX from CMCS in the case of single drug encapsulated system and 71% of 5-FU, 67% of DOX from CMCS in the case of dual drug loaded CMCS is observed. The release of the drugs is more sustained and prolonged when compared to the previous reports^26,36,37^.

#### Release profile of 5-FU@CMCS

The obtained release profile was split into 3 regions and fitted to the different kinetic models. Region 1 of the release profile from 1 to 4 h fitted well to the Higuchi kinetics, which explains the release of drug from a matrix and the diffusion coefficient value for this region is 0.11, showing Fickian diffusion to be responsible for drug release in this region. Region 2 from 5 to 35 h fits well to the first order kinetics which represents concentration dependent release. Here again Fickian diffusion is responsible for the release. Region 3 from 40 to 72 h fits well to the Korsmeyer-Peppas kinetics and the obtained diffusion coefficient value is less than 0.4 showing that Fickian diffusion causes the release of the drug in this region. So, it is observed that drug release is governed by diffusion in this system and also that diffusion is the predominant process occuring and not polymer chain relaxation.

#### Release profile of DOX@CMCS

The obtained release profile was split into two regions (region 1: 1 to 5 h and region 2: 15 to 72 h) both of which adhere to Korsmeyer-Peppas release kinetics, which specifically describes the release of drug from a polymer matrix. The diffusion coefficient values obtained for both the regions are less than 0.5, indicating that Fickian diffusion is responsible for the release of the drugs. Here again the release of drugs is mainly due to diffusion and not due to relaxation of polymer chain.

#### Release profile of 5-FU + DOX@CMCS

5-FU release. The release kinetics of 5-FU has been studied by splitting the obtained graph into 4 regions. Region 1 from 1 to 4 h fits well to first order kinetics and region 4 from 60 to 72 h adheres to Korsmeyer-Peppas kinetics. The diffusion coefficient value obtained for both the regions is greater than 0.5 but less than 0.9, indicating that non-Fickian diffusion or anomalous transport to be responsible for the release of the drug in these regions and the mechanism of drug release is governed by diffusion and swelling. The rate of diffusion and rate of swelling are comparable. The simultaneous occurrence of polymeric chain rearrangement and diffusion process cause the time-dependent anomalous effects. Region 2 from 5 to 25 h fits well to Higuchi kinetics and the region 3 from 35 to 50 h agrees well with the Hixson-Crowell kinetics. The diffusion coefficient for both the regions is less than 0.5, suggesting that Fickian diffusion causes the release of drugs in these regions.

##### DOX release

The phenomenon responsible for release of DOX has been analysed in the 3 regions of the release profile. Region 1 from 1 to 5 h fits well to Korsmeyer-Peppas kinetics and region 2 from 15 to 40 h adheres to first order kinetics. Fickian diffusion is speculated to be the mechanism causing the release of the drug as the obtained diffusion coefficient values are less than 0.5. Region 3 from 50 to 72 h adheres to Higuchi kinetics and the obtained diffusion coefficient value of 0.73, evidences non-Fickian diffusion or anamalous transport to be the reason behind the release of the drug in this region.

### *In-vitro* cytotoxicity studies

The cytotoxicity studies on MCF-7, carried out using drugs alone (Fig. 2(b)) and the prepared samples show a concentration dependent loss of viability. For the case of drugs alone (control) MTT experiment was carried out after 24 h of exposure and the cell viability is observed to be comparable to the values obtained on treatment of cells with the prepared samples for 48 h. This shows the ability of the samples to release the drugs in a controlled and sustained manner. 50% cell viability for the case of cells treated with the prepared samples was achieved on using 37.5 µg/ml, 40.9 µg/ml and 33.8 µg/ml of 5-FU@CMCS, DOX@CMCS and 5-FU+DOX@CMCS, respectively. The images of the cells at IC_50_ concentration is given as an inset in Fig. 3(b). Similarly, the inverted microscope image obtained after addition of 100 µg/ml of samples to the cells is shown in Fig. 3(c). The images of cells after treatment with dual drug loaded sample, obtained after AO-PI staining is shown in Fig. 3(d). The obtained results clearly indicate the effectiveness of drug loaded CMCS towards the inhibition of breast cancer cells. Among the 3 samples, dual drug loaded CMCS is seen to be more effective when compared to single drug loaded CMCS. All the samples show better cytotoxicity when compared to the previous reports^26,32,36,37^.

### Reactive Oxygen species (ROS)

ROS plays a pivotal role in deciding the condition of cells depending on the degree of oxidative stress. Undue production of ROS causes irrevocable damage to the DNA, proteins and lipids, thus leading to cell death through diverse means like apoptosis and necrosis^38,39^. The ROS levels in pretreated MCF-7 cells was determined by measuring the DHE fluorescence. The representative fluorescence images and plot are shown in Figs. S4 and 4(a), respectively. The ROS production is observed to be approximately 2.3 folds more for the case of cells treated with 5-FU+DOX@CMCS samples when compared to the control as well as MCF-7 cells treated with H_2_O_2_, showing the potential of the sample to induce oxidative stress which will lead to the destruction of carcinogenic cells.

**Figure 4:**
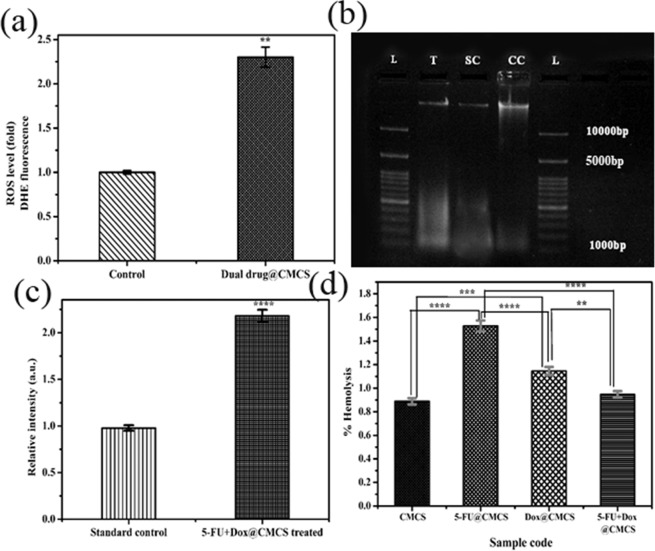
(**a**) Quantitative imaging analysis of ROS detected by DHE in live MCF-7 cells. Fluorescence intensity was assessed using ImageJ 1.47 v software (http://imagej.nih.gov/ij). Experiments are in triplicate and presented data are the mean ± s.e.m. **P < 0.01 (P = 0.0080) (student’s t-test), (**b**) Agrose Gel Electrophoresis demonstrating DNA fragmentation. MCF-7 cells treated with 15 µg/mL 5-FU+DOX@CMCS, which induces DNA fragmentation in 48 hours. Campothecin treated cells for 48 h served as the positive control (PC), (**c**) Comparison of relative intensity calculated from the different lanes agarose gel using ImageJ 1.47 v software (http://imagej.nih.gov/ij). Experiments are in triplicate and presented data are the mean ± s.e.m. ****P < 0.0001 (student’s t-test), (**d**) %Hemolysis exhibited by the various prepared samples. Values are expressed as Mean ± S.D. of 3 independent experiments. ****P < 0.0001, ***P = 0.0005, **P = 0.0027 (One way anova).

### DNA fragmentation

In order to demonstrate whether 5-FU and DOX encapsulated CMCS inhibits the proliferation of MCF-7 cells by instigating apoptosis, the cell death by DNA fragmentation was studied from 0 to 48 h (Figs. 4(b,c) & S6)^40,41^. The cells were treated with IC_50_ value of 5-FU+DOX@CMCS. The obtained results clearly indicate that the MCF-7 cells suffer apoptosis on treatment with 5-FU+DOX@CMCS. Presence of DNA fragments were clearly observed in the case of cells treated with the dual drug encapsulated sample while, parental DNA fragmentation was absent in the case of MCF-7 control. The relative intensity for the 5-FU+DOX@CMCS treated cells were approximately 2.2 times the value obtained for the standard control (camptothecin treated cells). This confirms that IC_50_ value of the prepared sample is enough to induce fragmentation and cause apoptosis in MCF-7 cells.

### Hemolysis

The hemocompatibility results obtained for the various samples are shown in Fig. 4(d). Hemolysis exhibited by all the samples are less than 5% which is well within the ASTM satandards. Thus, it is evident that all the samples are highly hemocompatible. The results obtained also elucidate the suitability of CMCS for the delivery of 5-FU and DOX separately as well as in combination. This is because of the reduction of side effects posed by the encapsulation of the anticancer drugs into CMCS, which is established from the highly hemocompatible nature of the samples.

## Discussions

Biopolymers have enthralled researchers due to their abundance, low cost and their extensive properties like biocompatibility, biodegradability and non-toxicity. It is these qualities which have made the biopolymers useful in various applications like food packaging, biosensing, water purification, wound healing, tissue regeneration, biomedical applications^42^ and drug delivery^12,19^. Among these, the capability of the biopolymers to enable the sustained and prolonged release of drugs has laid a basis for the treatment of grave diseases like cancer, using the already existent drugs. The use of these drugs has been limited, owing to their side effects. The encapsulation of these drugs into a biopolymer like chitosan and its derivatives will lead to a reduction in the number of dosages administered while simultaneously enabling pH sensitive delivery of the drugs.

In the present study, a water-soluble derivative of chitosan has been synthesized using the chemical method and used for the delivery of dual anticancer drugs namely 5-FU and DOX. The appearance of the various trade mark peaks of chitosan and the observance of splitting and shifting of peaks corresponding to amine and OH confirm the formation of N,O-carboxymethyl chitosan. Similarly, the FTIR results of drug encapsulated CMCS also shows the presence of new peaks, pertaining to the functional groups from the drugs. This is indicative of the encapulation of the drugs into CMCS. The presence of fluorine peaks in the EDX spectrum and the fluorescence emission and red fluorescence observed under the fluorescence microscope substantiate the incorporation of 5-FU and DOX into CMCS. Apart from these, the formation of CMCS and the encapsulation of drugs into it are evident from the observed zeta potential values, which also elucidate the colloidal stability of the drug encapsulated samples. The encapsulation efficiency of the drugs into CMCS has been calculated and it is >75% in all the cases. The release of the drugs from CMCS has been studied for 72 h and a sustained and prolonged release of the drugs is observed. A release of about 70% of 5-FU, 53% of DOX from CMCS in the case of single drug loaded system and 71% of 5-FU, 67% of DOX from CMCS in the case of dual drug loaded system is detected after 72 h. The cytotoxicity of the samples towards MCF-7 cells has been studied. The IC_50_ values are 16.5 µg/ml, 18.2 µg/ml and 14.9 µg/ml for 5-FU@CMCS, DOX@CMCS and 5-FU+DOX@CMCS, respectively. The obtained results clearly indicate the capability of the samples to inhibit the breast cancer cells. Among the three samples the dual drug encapsulated system is better as lower sample concentration is able to inhibit the carcinogenic cells. In order to understand the role of oxidative stress in the inhibition of MCF-7, ROS production was studied and the obtained results clearly indicate a 2.3-fold ROS production in the cells treated with dual drug encapsulated sample and H_2_O_2_ when compared to the cells treated with H_2_O_2_ alone. As the cell death due to increased concentration of ROS can be caused by various modes like apoptosis, necrosis, etc., DNA fragmentation was performed to study whether there is apoptosis. The obtained results clearly indicate that DNA fragmentation in cells treated with the prepared samples is high when compared to the standard control and also that the IC_50_ concentration itself caused DNA ladders. Thus, very low concentration of 5-FU+DOX@CMCS is able to effectively inhibit the carcinogenic cells by inducing oxidative stress and apoptosis due to the fragmentation of DNA. The hemolysis analysis results show the highly hemocompatible nature of the samples. This in turn is an evidence to the fact that the side effects caused by the drugs when used alone have been overcome to a certain extent by their encapsulation into CMCS. Therefore, the suitability of CMCS as a drug delivery carrier that can deliver dual anticancer drugs effectively, causing the destruction of breast cancer cells is manifested from the studies performed.

## Conclusion

An adept dual drug encapsulated biopolymer, exhibiting high cytotoxicity towards MCF-7 has been successfully synthesized using the chemical method. Carboxymethylation at both NH_2_ and OH sites of chitosan, leading to the formation of CMCS has been affirmed using FTIR. The encapsulation of 5-FU and DOX into CMCS have been confirmed from the EDX and fluorescence results. Encapsulation of the drugs into CMCS, causing a sustained and prolonged release when compared to the previous reports available for CMCS as well as chitosan based systems, showing the suitability of the system as a drug delivery carrier is evident from the obtained release profile. The current work clearly establishes the efficacy of dual drug loaded CMCS towards the inhibition of MCF-7 when compared to the CMCS containing either DOX or 5-FU alone as well as other systems reported previously, clearly highlighting the breast cancer cell repressing capability of the prepared samples. 33.8 µg/ml of dual drug loaded CMCS is able to cause the lysis of 50% of taken breast cancer cells. The highly hemocompatible nature of the synthesized samples, evidence the reduction in the side effects posed by the drugs, substantiating the efficacy of CMCS as a drug delivery carrier for the sustained and prolonged release of the used drugs.

## Materials and Methods

### Materials

Chitosan from crab shell, highly viscous was from sigma-Aldrich, glacial acetic acid from Merck, ethanol absolute 99.9% AR from Jebsen & Jessen GmbH & Co, Germany, Tripoly Phosphate (TPP) having >98% purity from sigma-Aldrich, Tween 80 for synthesis from Merck, 5-FU > 99% (HPLC) from sigma-Aldrich, Doxorubicin Hydrochloride 98.0–102.0% HPLC from sigma Aldrich, DMEM media from sigma Aldrich, Fetal Bovine Serum from Gibco, 0.25% Trypsinfrom Invitrogen and MTT from Sigma -Aldrich were used for the experiments. DMEM high glucose medium (#AL111, Himedia), Fetal Bovine Serum (#RM10432, Himedia), D-PBS (#TL1006, Himedia), Camptothecin-15 µM (Sigma), SDS 20% w/v (Himedia), Chloroform: isoamyl alcohol (24:1) (Himedia), Isopropanol (Himedia), Ethyl alcohol 70% v/v (Fisher Sceintific), Dihydroethidium (Hydroethidine) (Thermoscientific, Number: D-11347), DHE stock (5 mM, in DMSO), H_2_O_2_ (Fisher Scientific), Acridine orange - 20 µg/mL solution, Thermo Fischer, USA were used for the analyses. All experiments were carried out using triple distilled water.

### Methods

#### Synthesis of 5-FU@CMCS, DOX@CMCS and 5-FU+DOX@CMCS

Carboxymethyl chitosan was synthesized using the procedure followed elsewhere^43^. In brief, a solution containing chitosan (10 g) and NaOH (13.5 g) in ratio of 1:1.35 was prepared in 100 ml of water and allowed to swell for 2 h. To this, a solution containing 15 g of monochloroacetic acid in 20 ml of isopropanol was added. The reaction mixture was kept under stirring for 4 h after which the reaction was stopped by the addition of 200 ml of 70% ethanol. The solid was then desalted and dried at room temperature. The product obtained was suspended in 100 ml of 80% ethanol solution and 10 ml of 37% HCl, after which it was filtered and rinsed again in 75% ethanol and then dried to obtain carboxymethyl chitosan (CMCS) powder.

Drug loaded carboxymethyl chitosan was synthesized following the procedure reported previously^35^. Dual drug loaded carboxymethyl chitosan was synthesized as follows. Carboxymethyl chitosan solution was prepared such that the concentration of CMCS was 0.75 mg/mL. 5-FU solution (5-FU dissolved in water) containing 5 mg/ml of water and DOX solution containing 5 mg/ml of water were added into the chitosan solution one after the other. Tween 80 (0.5% (v/v)) was added to the above mixture. The dual drug containing CMCS solution was flush mixed with 1 mg/mL TPP solutions at a ratio of (2: 1) (v/v) (CMCS: TPP). The suspension was gently stirred for 3 h at room temperature to allow the adsorption of drugs on CMCS. The product obtained was then centrifuged, resuspended in water, freeze-dried and the powder obtained was used for further characterization.

#### Drug release studies

The release of the anticancer drugs from CMCS was studied using the dialysis bag technique, taking PBS solution having a pH ~5. The drug containing samples were suspended in 2 ml PBS and transferred into a dialysis bag (MWCO 1000 Da) and sealed. This was then submersed in 60 ml PBS maintained under stirring. At particular time intervals, 3 ml of PBS was collected from the beaker and analyzed using UV-Vis spectrophotometer. A calibration graph of absorbance Vs known drug concentration was plotted and used to calculate the concentration of released drug, which was further used to calculate the amount of drug released. The beaker was then replenished with 3 ml of fresh PBS after each sample collection^44,45^. The phenomenon responsible for the release of the drug in each portion of the release profile was analyzed by fitting the data to various kinetic models which are given below and the model which fitted best was chosen based on the obtained r^2^ values^46^.

##### Zero order kinetics

$${\rm{dM}}/{\rm{dt}}=\frac{{\rm{Ds}}}{{\rm{l}}}\times ({{\rm{C}}}_{{\rm{s}}}-{\rm{C}})$$

(or)

$$\frac{{\rm{dC}}}{{\rm{dt}}}=\frac{{\rm{DS}}}{{\rm{Vl}}}\times ({{\rm{C}}}_{{\rm{s}}}-{\rm{C}})$$

where,

M - Mass of the solute dissolved during time t

dM/dt - Velocity of mass dissolved (mass/time)

D - Coefficient of diffusion of solute in the solution

S - Solute area exposed

l - Thickness of the diffusion layer

C_s_ -Solubility of solid

C - Solute concentration in the solution at time t

dc/dt - Velocity of dissolution and V is the volume of solution.

##### First order kinetics

$$\log \,{{\rm{Q}}}_{1}=\,\log \,{{\rm{Q}}}_{0}+\,\log \,{{\rm{k}}}_{1}{\rm{t}}/2.303$$

here,

Q_1_ - Amount of drug released in time t

Q_0_ - Initial concentration of drug dissolved

K_1_ - First-order constant.

##### Higuchi kinetics

$${{\rm{f}}}_{1}={\rm{Q}}={{\rm{K}}}_{{\rm{H}}}\sqrt{{\rm{t}}}$$

This equation represents a linear function that relates the concentration of drug to the √time. K_H_ is the Higuchi release constant.

##### Hixson-crowell kinetics

$$\sqrt[3]{1-{{\rm{f}}}_{{\rm{i}}}}=1-{{\rm{K}}}_{{\rm{\beta }}}{\rm{t}}$$

where,

f_1_ = 1 − (W_i_/W_0_) - Fraction of drug dissolved in time t

Kβ - Release constant

The above equation relates the cube root of the remaining fraction of drug that is not released with the time.

##### Korsmeyer-peppas kinetics:

$$\frac{\log \,{{\rm{M}}}_{({\rm{i}}-1)}}{{{\rm{M}}}_{\infty }}=\,\log \,{\rm{K}}+{\rm{N}}\,\log ({\rm{t}}-1)$$

where,

M_∞_ - Amount of drug at the equilibrium state

M_i_ - Amount of drug released in time t

K - Release velocity constant

n - Exponent of release (related to the drug release mechanism) in function of time t or the diffusion coefficient and l is the latency time.

#### Cell lines and culture conditions

MCF-7 (Human Breast Adenocarcinoma) cells were procured from National Centre for Cell Sciences (NCCS), Pune, India and maintained in Dulbecco’s modified Eagles medium, DMEM. DMEM supplemented with 10% FBS, L-glutamine, sodium bicarbonate and antibiotic solution containing: Penicillin (100 µg/ml), Streptomycin (100 µg/ml), and Amphoteracin B (2.5 µg/ml) was used to culture the obtained cell line in a 25 cm^2^ tissue culture flask. The cells were then incubated at 37 °C in a humidified 5% CO_2_ incubator and the cell viability was obtained by the MTT assay method as well as by observing the cells directly under a inverted phase contrast microscope.

##### Cells seeding in 96 well plate

Confluent, two-day old cell monolayer was trypsinized and the cells were suspended in 10% growth medium. 100 µl cell suspension (5 × 10^4^ cells/well) was seeded in a 96 well tissue culture plate and incubated at 37 °C in 5% CO_2_ incubator.

##### Compound stock preparation

A cyclomixer was used to suspend 1 mg of the prepared samples in 1 ml DMEM, which was subsequently filtered using a 0.22 µm Millipore syringe filter to ensure the sterility.

##### Anticancer activity evaluation

The growth medium was removed after 24 h and the drug containing samples (5-FU@CMCS, DOX @CMCS and 5-FU+DOX @CMCS) suspended in 5% DMEM were five times serially diluted using two-fold dilution (100 µg, 50 µg, 25 µg, 12.5 µg, 6.25 µg in 500 µl of 5% DMEM) and 100 µl of each concentration was added in triplicates to the respective wells and incubated at 37 °C. Untreated control cells were also maintained.

##### Anticancer assay by direct microscopic observation

The entire plate was observed using an inverted phase contrast tissue culture microscope (Olympus CKX41 with Optika Pro5 CCD camera) after 24 hours and the observations were recorded in the form of images. The changes in the cell morphology such as rounding or shrinking of cells, granulation and vacuolization in the cytoplasm of the cells, were used as the indicators showing the cytotoxicity of the samples.

##### Anticancer assay by MTT method

Fifteen milligrams of MTT was reconstituted in 3 ml PBS till it dissolved completely. This was then sterilized by filter sterilization. The samples added to the wells were removed after 24 h of incubation, followed by the addition of 30 µl of reconstituted MTT solution to all test and cell control wells. The plate was gently shaken and then incubated for 4 h at 37 °C in humidified 5% CO_2_ incubator. After the incubation period, the supernatant was removed and 100 µl of MTT Solubilization Solution (Dimethyl sulphoxide, DMSO, Sigma Aldrich, USA) was added to the wells. This was mixed gently by pipetting up and down in order to solubilize the formazan crystals. A microplate reader was used to measure the absorbance values at a wavelength of 540 nm^47,48^.

The percentage of growth inhibition was calculated using the formula:

$$ \% \,{\rm{of}}\,{\rm{viability}}=\frac{{\rm{Mean}}\,{\rm{OD}}\,{\rm{of}}\,{\rm{sample}}}{{\rm{Mean}}\,{\rm{OD}}\,{\rm{of}}\,{\rm{control}}\,{\rm{group}}}\times 100$$

and also, by performing a non linear dose responsive fit to the obtained data (Fig. S3).

#### Acridine orange and ethidium bromide staining

MCF-7 cells were cultured in a 6-well plate above sterile coverslips coated with Poly L Ornithine solution at a density of 2 × 10^5^ cells/2 ml and incubated in a CO_2_ incubator at 37 °C for 24 hours. The spent medium was aspirated and the cells were treated with required concentration (IC_50_ value) of experimental compounds (5-FU+DOX@CMCS) and controls, in 2 ml of culture medium and incubated for 48 hours. At the end of the treatment, the medium was removed from all the wells and the cells were washed with PBS. The coverslips were removed from the six well plate and washed with 1 ml of 1X DPBS. The cells were then stained with 200 µL staining solution for 10 min. After this, the staining solution was removed and a drop of mounting medium was added before imaging. The cells were then observed under fluorescence microscope with filter cube with Excitation 560/40 nm and Emission 645/75 nm for EtBr and Excitation 470/40 and Emission 525/50 for Acridine orange. Images were overlayed using ImageJ Software 1.47 v.

#### ROS study by DHE staining

Cells were cultured in a 6-well plate at a density of 3 × 10^5^ cells/2 ml and incubated in CO_2_ incubator at 37 °C for 24 hours. The spent medium was aspirated and the cells were washed with 1 ml of 1X PBS. The cells were then treated with required different concentrations of experimental compounds (14.9 µg of 5-FU+DOX@CMCS) and control in 2 ml of culture medium and incubated for 48 hours. One of the wells was left untreated to be used as negative control. The medium was aspirated and the cells were washed with 1 ml 1X PBS. The cells were then treated with required concentration of toxic compound (H_2_O_2_–200 µM) in 2 ml of culture medium and incubated for 4 hours. At the end of the treatment, the medium was removed from all the wells and PBS wash was given. The coverslips were removed from the six well plate and washed with 1 ml of 1X DPBS. The cells were stained with 1000 µL of DHE Stain (5 mM Stock) for 10 min. The staining solution was removed and a drop of mounting medium was added before imaging. After this, the cells were observed and captured under fluorescence microscope with filter cube with Excitation 5618/30 nm and Emission 606/25 nm for DHE Stain. The fluorescence intensity was then measured using ImageJ 1.47 v software (http.//imagej.nih.gov/ij) and then a graph of ROS (fold) production was plotted^39^.

#### DNA fragmentation (Gel Electrophoresis)

0.5 × 10^6^ cells/1 ml were cultured in a 6-well plate and incubated for 24 h in a CO_2_ incubator at 37 °C. The spent medium was removed and the cells were treated with required concentration of 5-FU+DOX@CMCS in 2 ml of culture medium and incubated for 24 hours. Controls were also maintained separately. After the treatment, the medium was aspirated from all the wells and the cells were washed with PBS. The pellet was then collected.

1. DNA Isolation from cell lines treated with samples:
  The pellet obtained by centrifuging at 1000 rpm, 20 min. at 4 °C was washed with sterile distilled water. 675 μl of extraction buffer was added to the cells after which they were incubated at 37 °C for 30 min. This was followed by the addition of 75 μl of SDS (20%) and incubation at 65 °C for 2 hours. The cells were then centrifuged at 10000 rpm for 10 min at 4 °C. The obtained clear solution was collected in a sterile microcentrifuge tube. To this, equal volume of chloroform: isoamyl alcohol (24:1) was added and then centrifugation was done at 10000 rpm for 10 min. at 4 °C. The obtained aqueous phase was removed and transferred to a sterile microcentrifuge tube. Isopropyl alcohol (0.6 times the obtained volume) was added and the cells were incubated at room temperature for 1 h followed by centrifugation at 10000 rpm for 10 min. The obtained pellet was washed in 500 μl of 70% ethanol and centrifuged at 10000 rpm for 10 min at room temperature. The pellet was dried and subsequently dissolved in 20 μl of sterile distilled water.
2. Gel Electrophoresis:
  0.8 g agarose powder was weighed and taken in a conical flask. To this, 100 ml of TAE Buffer was added and mixed properly. The agarose was melted in a microwave/hot water bath till a clear solution was obtained. This was allowed to cool to about 50–55 °C, by swirling the flask. After this, 5 µl of ethidium bromide solution was added to the agarose gel and mixed well. The ends of the casting tray were sealed with two layers of tape and the combs were placed in the gel casting tray. The agarose solution was poured into the casting tray and was let to solidify. The combs were then pulled out, after which the tapes were removed. The gel was placed in the electrophoresis chamber. Sufficient quantity of TAE Buffer was added such that there is about 2–3 mm of buffer over the gel. 6 μl of 6X sample loading buffer was added to each 25 μl PCR reaction. Each sample/ DNA ladder was pipetted into separate wells in the gel. The electrode wires were connected to the power supply which was set to about 100 V. Once the samples had run sufficiently, the power supply was turned off and the gel was removed using gloves and visualized under U.V. light. UV trans-illuminator was then used to photograph and document the results. The intensity value of each lane was measured using ImageJ 1.47 v software (http.//imagej.nih.gov/ij) software and the relative intensity was calculated^49,50^.

#### Hemolysis

Blood was collected from healthy donors (HR/2018/MS/003) and transferred to heparin tubes to prevent clotting. The collected blood was subsequently diluted using saline to obtain UV-Vis absorbance less than 1. A specific weight of prepared samples was taken and to which 250 µl of diluted blood was added to and incubated for 20 mins. This was followed by the addition of 2 mL of saline to stop the reaction and left undisturbed for 1 hr. Diluted blood along with 2 mL of saline in the absence of sample served as negative control. Diluted blood with deionized water served as positive control. The supernatant was collected after centrifugation and the absorbance was measured using Jasco V-760 UV-Visible spectrometer at 545 nm^51^.

Percentage of hemolysis was calculated using the formula

$$ \% \,{\rm{Hemolysis}}=\frac{{\rm{OD}}\,{\rm{of}}\,{\rm{the}}\,{\rm{test}}\,{\rm{sample}}-{\rm{OD}}\,{\rm{of}}\,{\rm{the}}\,{\rm{negative}}\,{\rm{control}}}{{\rm{OD}}\,{\rm{of}}\,{\rm{the}}\,{\rm{positive}}\,{\rm{control}}-{\rm{OD}}\,{\rm{of}}\,{\rm{the}}\,{\rm{negative}}\,{\rm{control}}}$$

The hemolysis percentage obtained for the case of all the prepared samples were compared with ASTM standard which states that <5% hemolysis represents highly hemocompatible nature, hemolysis within 10% depicts hemocompatibility and >20% hemolysis describes the non-hemocompatible nature of samples^52^.

All methods involving the human participants were carried out in accordance with the ethical principles of the Declaration of Helsinki and informed consent was obtained from all participants.

### Instrumentation details

The crystal structure of the prepared samples was analyzed using X-ray diffractometer (XPERT-PRO, stage PW3071) using Cu Kα (1.5406 Å) radiation in the two-theta range of 5° to 80°. Jasco Fourier transform infrared spectrometer (FTIR-6300) with KBr pellet technique was used to identify the functional groups of all the samples in the wavenumber range of 4000–400 cm^−1^ in transmission mode (64 scans for each samples) and UV spectrum was recorded at room temperature using Jasco V760 UV-VIS (double beam) instrument. Dynamic light scattering (DLS, Malvern Zetasizer Nano-ZS) technique was used to measure the Zeta potential of the prepared samples. He-Ne diode laser having a wavelength of 633 nm was used to scatter the particles at room temperature at a fixed angle of 90°. Powder sample (less than 1 mg) was dispersed for 1 h in deionized water using a probe sonicator. After sonication, the solution containing the dispersed (1.5 mL) particles was carefully transferred to a Malvern universal cell cuvette and then the experiment was performed in triplicates. SEM was carried out with a resolution of 3.0 nm at 30 kV using Carl Zeiss MA15/EVO 18. A small amount of sample was spread on the surface of a carbon tape and then the sample surface was sputtered using gold using ion sputter coating before imaging. Fluoromax 2 and Leica DM 2500 was used for steady state fluorescence measurements, using red filter. Olympus Phase Contrast Microscope, JAPAN with Optika Pro 5 Camera at 10X magnification and ERBA ELISA Reader, GERMANY were used for cytotoxicity studies. Eppendorf CO_2_ Incubator, Germany was used for cell culture studies^35^.

### Ethical statement

The authors confirm that the ethical policies of the journal have been adhered to and the appropriate institutional ethical review committee (Global hospitals) approval has been received. Project No. HR/2018/ MS/003. Project Title: *In Vitro* hepatic differentiation of human adult stem cells. Project Investigators: Prof. Mohammed Rela, Dr. Mettu Srinivas Reddy, Ms. R. Subathra and Ms. Catherin Ann Martin dated: January 1, 2018.

## Supplementary information

Supplementary information.
