# Supplementary material for: A competent bidrug loaded water soluble chitosan derivative for the effective inhibition of breast cancer
Source: Sci Rep. 2020 Mar 4;10:3991. doi: 10.1038/s41598-020-60888-5 (PMC7055325; doi:10.1038/s41598-020-60888-5)
Supplement: Supplementary file 1 — Supplementary information. [file 41598_2020_60888_MOESM1_ESM.docx]

**A competent bidrug loaded water soluble chitosan derivative for the effective inhibition of breast cancer**

E. A. K. Nivethaa^a^, S. Baskar^a^, Catherine Ann Martin^a^, J. Ramana Ramya^d^, A. Stephen^b^,
V. Narayanan^c^, B.S. Lakshmi^f^, Olga V. Frank-Kamenetskaya^e^, R. Subathra^g^,
S. Narayana Kalkura^a.*^

*^a^ Crystal Growth Centre, Anna University, Chennai – 600025, India.*

*^b^Department of Nuclear Physics, University of Madras, Chennai – 600025, India.*

*^c^Department of Inorganic Chemistry, University of Madras, Chennai – 600025, India.*

*^d^National Centre for Nanoscience and Nanotechnology, University of Madras, Chennai – 600025, India.*

*^e^Department of Crystallography, Institute of Earth Sciences, St. Petersburg State University, Russia -199034*

*^f^ Centre for Biotechnology, Anna University, Chennai – 600025, India.*

*^g^National foundation for liver research, Global hospitals, Perumbakkam, Chennai -600100, India*

**Corresponding Author: Tel. No: 91-44-22358335,*

*Email address:* [*kalkura@yahoo.com*](mailto:kalkura@yahoo.com)*.*


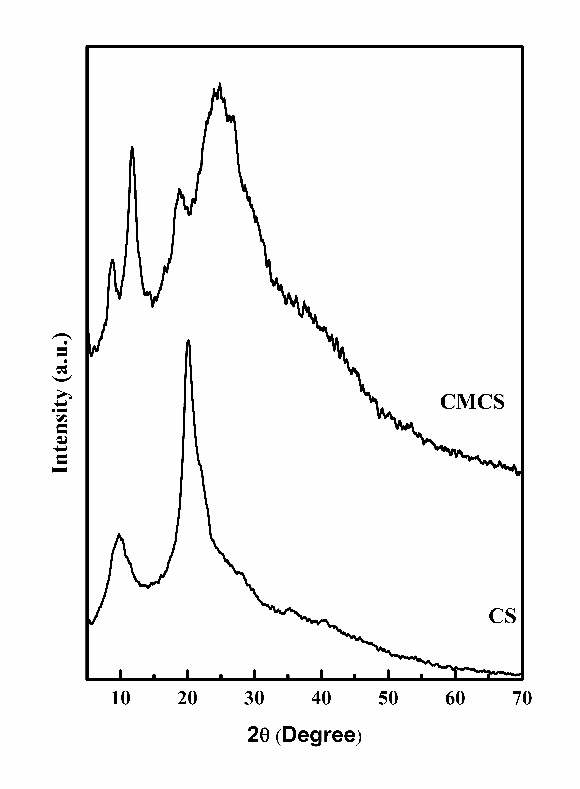
 **X-Ray diffraction analysis**

**Figure S1.** XRD pattern of chitosan and carboxymethyl chitosan

The XRD pattern of chitosan and CMCS are shown in figure S1. The obtained patterns show the semi crystalline nature of both polymers. The XRD pattern of CS shows the presence of two characteristic peaks at 2θ ~ 10⁰ and 20⁰ whereas, the XRD pattern of CMCS shows the presence of peaks at 2θ ~ 9.5⁰, 12.5⁰, 19.6⁰, 24⁰ which is in good agreement with the peaks reported for CMCS. A shift and reduction in the intensity of the peaks are observed for the case of CMCS when compared to CS.

The SEM images of CMCS as well as drug loaded CMCS are shown in figure S2. The SEM image of CMCS evidences fused spheres forming a matrix like structure. The image obtained for 5-FU encapsulated CMCS shows the formation of a porous matrix due to the crosslinking of polymer. The SEM image of dox@CMCS shows the formation of a polymer matrix structure in which uniform geometric doxorubicin crystals are housed on the polymer matrix. The SEM image obtained for dual drug loaded CMCS is a blend of the SEM images of 5-FU@CMCS and dox@CMCS. The morphology of the dual drug loaded system bears close semblance to a lava bed.


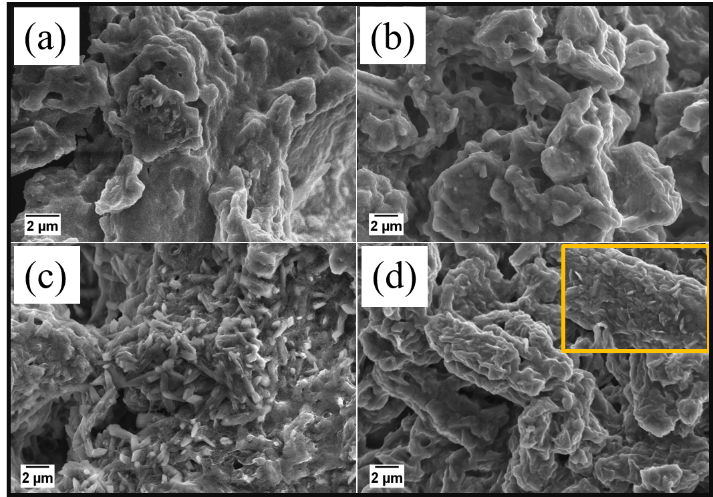


**Figure S2.** SEM image of (a) CMCS, (b) 5-FU@CMCS, (c) dox@CMCS and (d) 5-FU + dox@CMCS with a magnified image as inset

**cells+sample+H_2_O_2_**


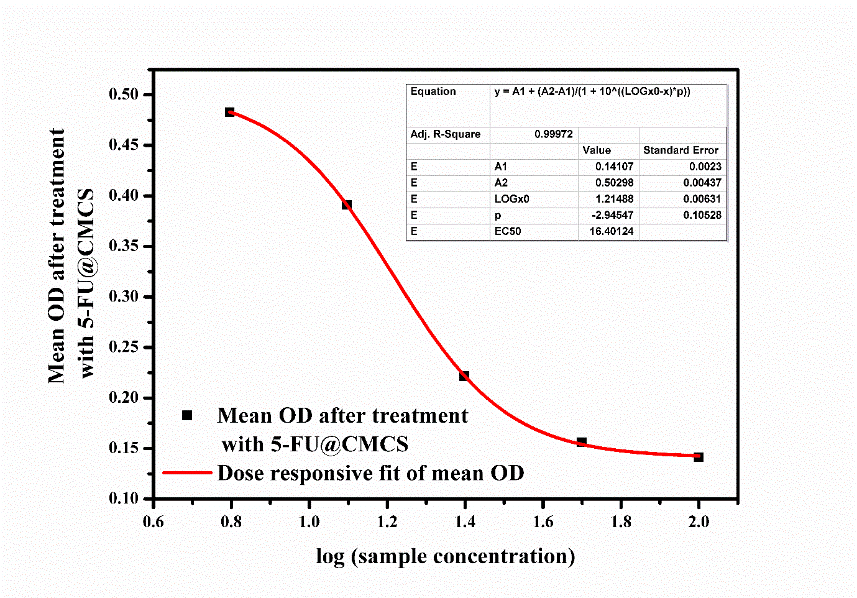

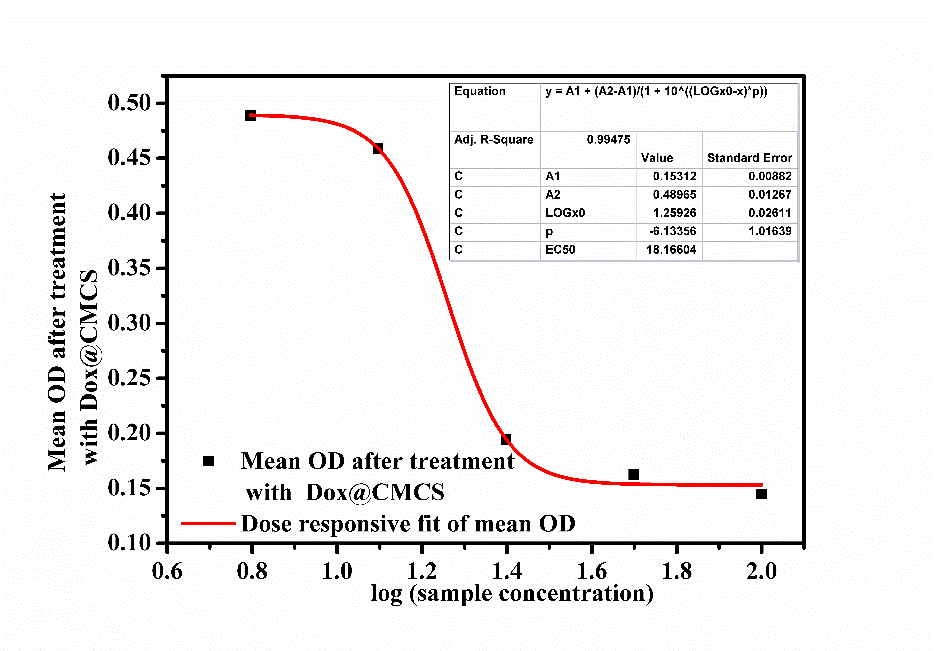

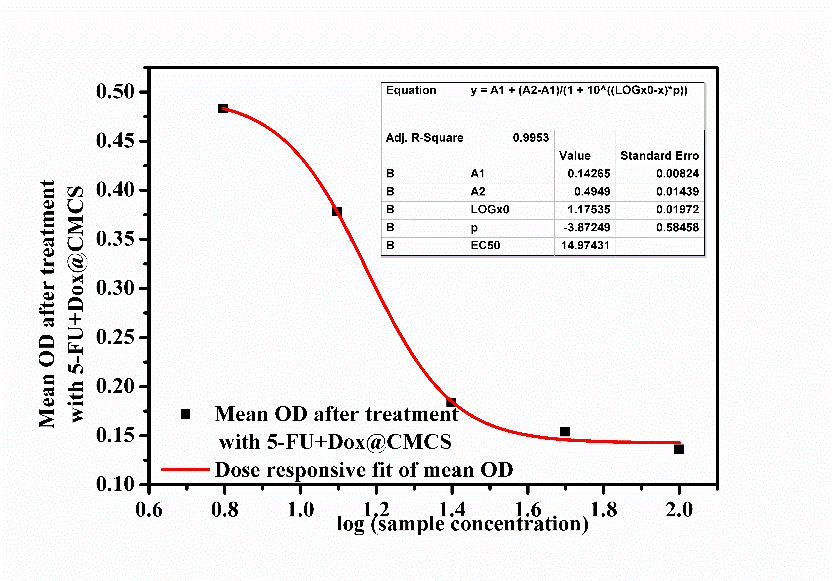


**Figure S3.** Non-linear dose response fit for the calculation of IC_50_


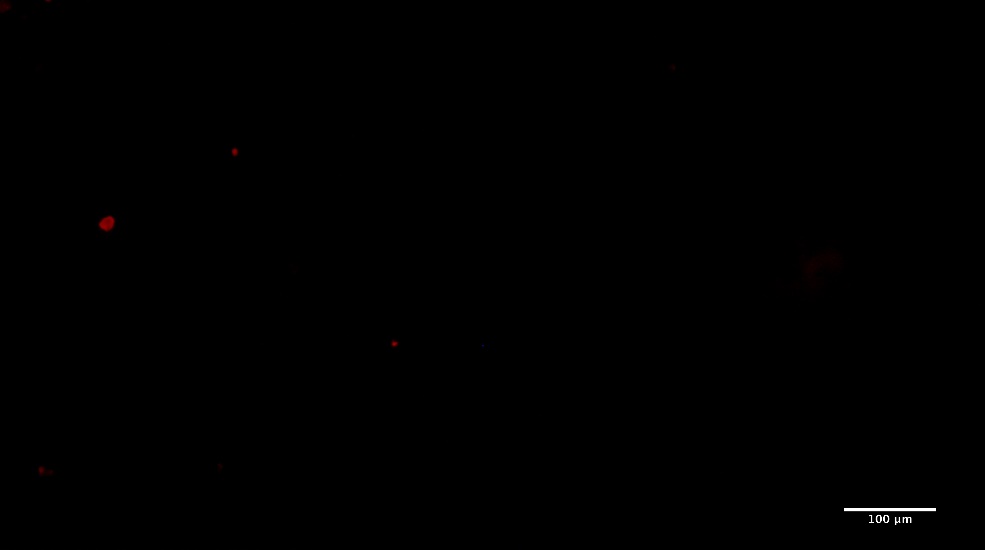

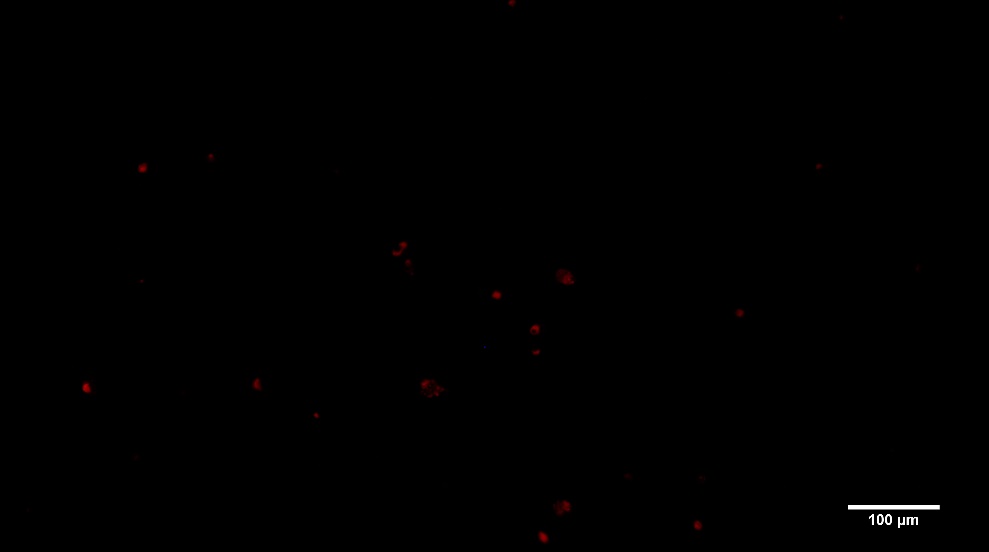

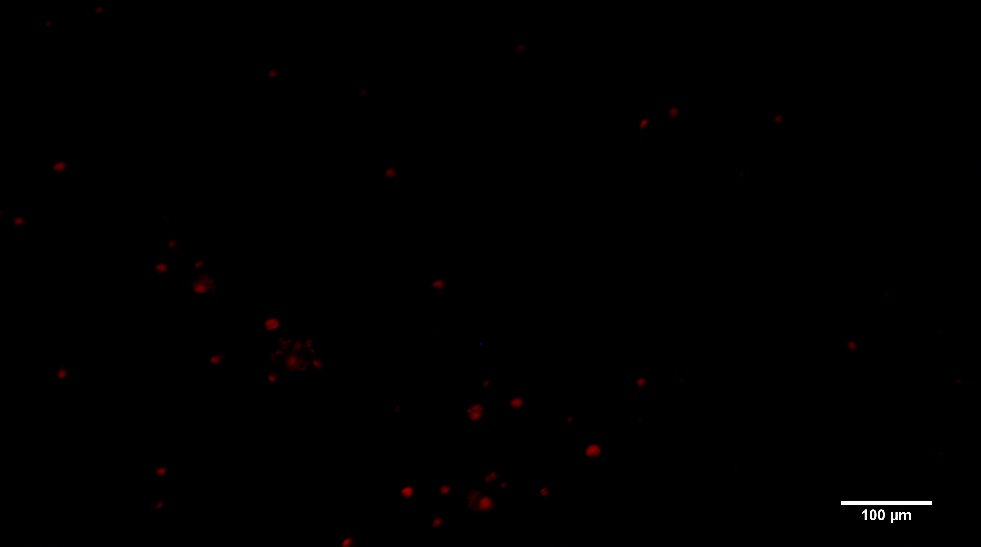


**control**

**cells+H_2_O_2_**

**cells+samples+H_2_O_2_**

**Figure S4.** Fluorescence microscope images for ROS production


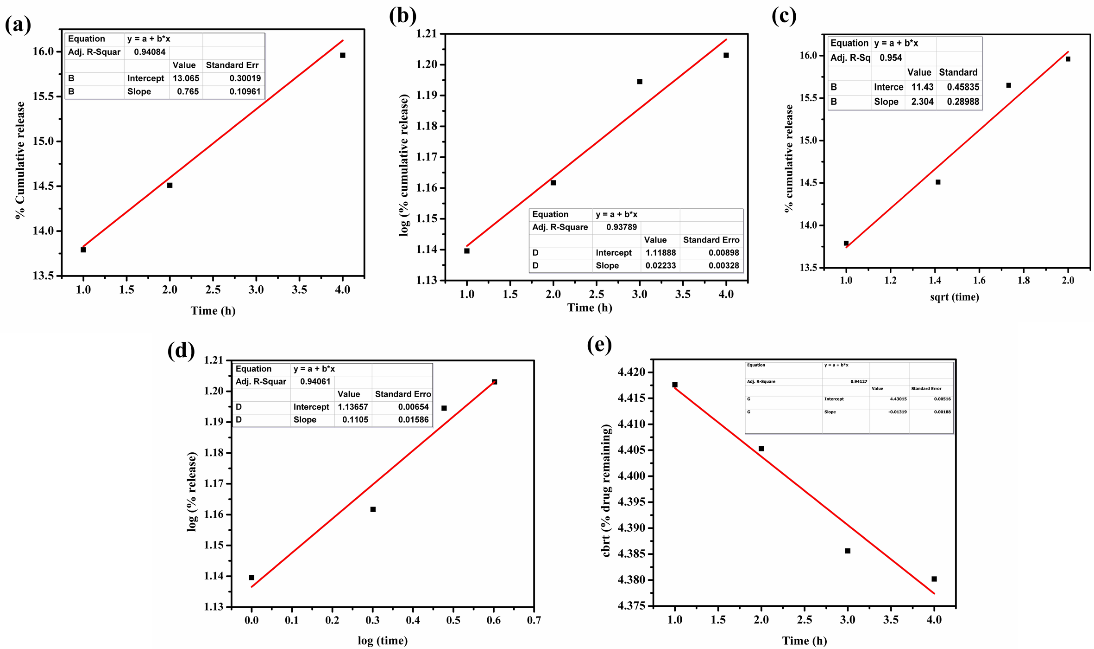


**Figure S5.** Kinetic model fitting of the first region of the release profile of 5-FU@CMCS showing (a) Zero order kinetics, (b) first order kinetics, (c) Higuchi kinetics, (d) Korsmeyer-Peppas kinetics and (e) Hixson-Crowell kinetics


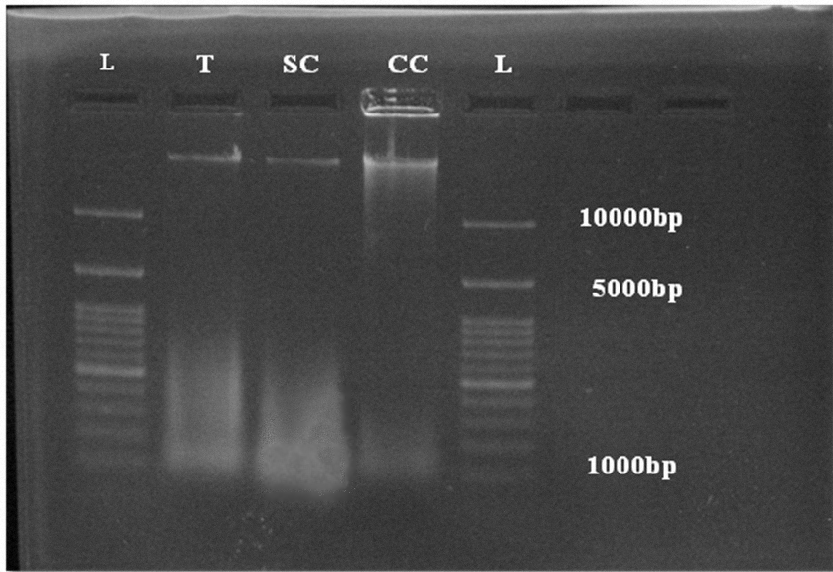


**Figure S6.** Original full length Agrose Gel Electrophoresis demonstrating DNA fragmentation.
MCF-7 cells treated with 15µg/mL 5-FU+DOX@CMCS, which induces DNA fragmentation in 48 hours. Campothecin treated cells for 48 h served as the positive control.
